# Supplementary material for: Genome-wide study of a Neolithic Wartberg grave community reveals distinct HLA variation and hunter-gatherer ancestry
Source: Commun Biol. 2021 Jan 25;4:113. doi: 10.1038/s42003-020-01627-4 (PMC7835224; doi:10.1038/s42003-020-01627-4)
Supplement: Supplementary file 2 — Supplementary Information [file 42003_2020_1627_MOESM2_ESM.pdf]

Supplementary Information

Genome-wide study of a Neolithic Wartberg grave community reveals distinct HLA variation and hunter-gatherer ancestry

Alexander Immel, Federica Pierini, Christoph Rinne, John Meadows, Rodrigo Barquera, András Szolek, Julian Susat, Lisa Böhme, Janina Dose, Joanna Bonczarowska, Clara Drummer, Katharina Fuchs, David Ellinghaus, Jan Christian Kässens, Martin Furholt, Oliver Kohlbacher, Sabine Schade-Lindig, Andre Franke, Stefan Schreiber, Johannes Krause, Johannes Müller, Tobias L. Lenz, Almut Nebel, Ben Krause-Kyora

Radiocarbon dating

Twenty-five of the aDNA samples were dated by radiocarbon (^{14}C) at the Leibniz-Laboratory for AMS Dating and Stable Isotope Research, Kiel University, Kiel, Germany. Bone was crushed, ultrasonicated in acetone, and rinsed repeatedly in demineralized water, before collagen was extracted[1]: bone was demineralized at room temperature in 1% HCl, rinsed and then treated with 1% NaOH (1 h, 60°C) to dissolve secondary organic compounds, rinsed again and re-acidified. The resulting collagen was dissolved in a weakly acidic solution (14 h, 72° C) and filtered through pre-baked 4.5 µm silver filters, before freeze-drying. In a sealed quartz tube, some collagen was combusted to CO_2, which was reduced to graphite and measured by Accelerator Mass Spectrometry (AMS)[2,3]. Collagen %C, %N, and the dietary stable isotopes $\delta^{13}C$ and $\delta^{15}N$ were measured by Elemental Analysis-Isotope Ratio Mass Spectrometry (EA-IRMS) at isolab GmbH, Schweitenkirchen, Germany[4].

All samples gave good yields (>1% by weight) of collagen whose atomic C/N ratio was close to the expected value (3.16–3.32)[5]. These data support the validity of the ^{14}C results (Supplementary Table 1). The ^{14}C ages, although tightly clustered, are not statistically consistent with a single ^{14}C age (T=51.1, T'(5%)=36.4, df=24)[6], so it is unlikely that these 25 individuals were exactly contemporaneous.

Human ^{14}C ages can be misleadingly old, due to consumption of fish and other aquatic taxa ('dietary ^{14}C reservoir effects')[7]. The tight clustering of ^{14}C ages is difficult to reconcile with significant reservoir effects, however, and ^{14}C ages appear to be independent of $\delta^{13}C$ and $\delta^{15}N$ (Supplementary Fig. 1). The $\delta^{13}C$ values are all compatible with terrestrial diets, based on the C_3 photosynthetic pathway. Some $\delta^{15}N$ values are relatively high (> 12 ‰), but in these cases the bone sampled was the pars petrosa, which undergoes little remodeling over a person's lifetime; petrous bone $\delta^{13}C$ and $\delta^{15}N$ values therefore reflect diet in early childhood[8]. $\delta^{15}N$

variation among petrous bone samples probably reflects (in part) differences in weaning age. Moreover, petrous bone and dentine collagen forms over perhaps 2-3 years at most, whereas $\delta^{13}C$ and $\delta^{15}N$ values from adult crania (samples KH150203, KH150287, KH150288, KH150289) represent average diets over the last 1-2 decades of life. These four samples, which gave some of the highest ^{14}C ages, appear to represent almost identical, terrestrial-based diets (Supplementary Fig. 1). We therefore believe that the risk of significant dietary reservoir effects is negligible.

Individually, the ^{14}C ages date all 25 burials to the calibration plateau spanning the last third of the 4th millennium cal BC[9,10]. Supplementary Fig. 2 uses a kernel-density estimation model to summarize the dates[11], which suggests that most, if not all individuals, could date to the 33rd century (3300–3200 cal BC).

Admixture dating

ALDER linkage disequilibrium decay rates for Anatolia Neolithic and Koros Hungary EN HG:

---- fit on data from 1.00 to 50.00 cM (unconstrained affine term) ----
d>1.00 decay: 20.80 +/- 4.03 z = 5.16 *
d>1.00 amp_tot: 0.00018393 +/- 0.00002653
d>1.00 amp_exp: 0.00017777 +/- 0.00002584 z = 6.88 *
d>1.00 amp_aff: 0.00001231 +/- 0.00000353

---- fit on data from 1.10 to 50.00 cM (unconstrained affine term) ----
d>1.10 decay: 19.38 +/- 3.94 z = 4.92 *
d>1.10 amp_tot: 0.00017330 +/- 0.00002605
d>1.10 amp_exp: 0.00016738 +/- 0.00002536 z = 6.60 *
d>1.10 amp_aff: 0.00001184 +/- 0.00000357

---- fit on data from 1.20 to 50.00 cM (unconstrained affine term) ----
d>1.20 decay: 19.27 +/- 4.09 z = 4.71 *
d>1.20 amp_tot: 0.00017241 +/- 0.00002681
d>1.20 amp_exp: 0.00016651 +/- 0.00002628 z = 6.33 *
d>1.20 amp_aff: 0.00001180 +/- 0.00000346

---- fit on data from 1.30 to 50.00 cM (unconstrained affine term) ----
d>1.30 decay: 18.80 +/- 4.11 z = 4.58 *
d>1.30 amp_tot: 0.00016879 +/- 0.00002769
d>1.30 amp_exp: 0.00016298 +/- 0.00002711 z = 6.01 *
d>1.30 amp_aff: 0.00001163 +/- 0.00000351

---- fit on data from 1.40 to 50.00 cM (unconstrained affine term) ----
d>1.40 decay: 19.41 +/- 4.53 z = 4.29 *
d>1.40 amp_tot: 0.00017358 +/- 0.00003022
d>1.40 amp_exp: 0.00016765 +/- 0.00002951 z = 5.68 *
d>1.40 amp_aff: 0.00001185 +/- 0.00000359

ALDER linkage disequilibrium decay rates for Anatolia Neolithic and Bichon HG:

---- fit on data from 1.00 to 50.00 cM (unconstrained affine term) ----
d>1.00 decay: 19.00 +/- 3.40 z = 5.59 *
d>1.00 amp_tot: 0.00023442 +/- 0.00002464
d>1.00 amp_exp: 0.00022666 +/- 0.00002401 z = 9.44 *
d>1.00 amp_aff: 0.00001551 +/- 0.00000484

---- fit on data from 1.10 to 50.00 cM (unconstrained affine term) ----
d>1.10 decay: 18.49 +/- 3.31 z = 5.58 *
d>1.10 amp_tot: 0.00022908 +/- 0.00002497
d>1.10 amp_exp: 0.00022145 +/- 0.00002432 z = 9.11 *
d>1.10 amp_aff: 0.00001526 +/- 0.00000486

---- fit on data from 1.20 to 50.00 cM (unconstrained affine term) ----
d>1.20 decay: 18.11 +/- 3.43 z = 5.27 *
d>1.20 amp_tot: 0.00022495 +/- 0.00002699
d>1.20 amp_exp: 0.00021742 +/- 0.00002637 z = 8.24 *
d>1.20 amp_aff: 0.00001506 +/- 0.00000486

---- fit on data from 1.30 to 50.00 cM (unconstrained affine term) ----
d>1.30 decay: 17.42 +/- 3.42 z = 5.09 *

d>1.30 amp_tot: 0.00021747 +/- 0.00002751
d>1.30 amp_exp: 0.00021012 +/- 0.00002682 z = 7.83 *
d>1.30 amp_aff: 0.00001470 +/- 0.00000492

---- fit on data from 1.40 to 50.00 cM (unconstrained affine term) ----
d>1.40 decay: 16.86 +/- 3.45 z = 4.89 *
d>1.40 amp_tot: 0.00021114 +/- 0.00002780
d>1.40 amp_exp: 0.00020394 +/- 0.00002691 z = 7.58 *
d>1.40 amp_aff: 0.00001440 +/- 0.00000505

ALDER linkage disequilibrium decay rates for Anatolia_Neolithic and Serbia_HG:

---- fit on data from 1.00 to 50.00 cM (unconstrained affine term) ----
d>1.00 decay: 15.31 +/- 2.43 z = 6.29 *
d>1.00 amp_tot: 0.00013279 +/- 0.00001396
d>1.00 amp_exp: 0.00012896 +/- 0.00001392 z = 9.27 *
d>1.00 amp_aff: 0.00000766 +/- 0.00000375

---- fit on data from 1.10 to 50.00 cM (unconstrained affine term) ----
d>1.10 decay: 14.74 +/- 2.46 z = 5.99 *
d>1.10 amp_tot: 0.00012882 +/- 0.00001344
d>1.10 amp_exp: 0.00012513 +/- 0.00001338 z = 9.35 *
d>1.10 amp_aff: 0.00000738 +/- 0.00000379

---- fit on data from 1.20 to 50.00 cM (unconstrained affine term) ----
d>1.20 decay: 14.54 +/- 2.62 z = 5.55 *
d>1.20 amp_tot: 0.00012745 +/- 0.00001392
d>1.20 amp_exp: 0.00012381 +/- 0.00001374 z = 9.01 *
d>1.20 amp_aff: 0.00000728 +/- 0.00000386

---- fit on data from 1.30 to 50.00 cM (unconstrained affine term) ----
d>1.30 decay: 14.24 +/- 2.73 z = 5.21 *
d>1.30 amp_tot: 0.00012531 +/- 0.00001454
d>1.30 amp_exp: 0.00012174 +/- 0.00001420 z = 8.57 *
d>1.30 amp_aff: 0.00000713 +/- 0.00000396

---- fit on data from 1.40 to 50.00 cM (unconstrained affine term) ----
d>1.40 decay: 14.36 +/- 2.86 z = 5.02 *
d>1.40 amp_tot: 0.00012615 +/- 0.00001614
d>1.40 amp_exp: 0.00012256 +/- 0.00001578 z = 7.77 *
d>1.40 amp_aff: 0.00000719 +/- 0.00000396

ALDER linkage disequilibrium decay rates for Anatolia_Neolithic and Iron_Gates_HG:

---- fit on data from 1.20 to 50.00 cM (unconstrained affine term) ----
d>1.20 decay: 14.44 +/- 1.65 z = 8.78 *
d>1.20 amp_tot: 0.00013828 +/- 0.00000954
d>1.20 amp_exp: 0.00013465 +/- 0.00000932 z = 14.45 *
d>1.20 amp_aff: 0.00000726 +/- 0.00000216

---- fit on data from 1.30 to 50.00 cM (unconstrained affine term) ----
d>1.30 decay: 13.96 +/- 1.64 z = 8.51 *
d>1.30 amp_tot: 0.00013466 +/- 0.00000977
d>1.30 amp_exp: 0.00013117 +/- 0.00000953 z = 13.77 *
d>1.30 amp_aff: 0.00000697 +/- 0.00000219

---- fit on data from 1.40 to 50.00 cM (unconstrained affine term) ----
d>1.40 decay: 13.77 +/- 1.66 z = 8.32 *
d>1.40 amp_tot: 0.00013319 +/- 0.00001000
d>1.40 amp_exp: 0.00012976 +/- 0.00000973 z = 13.34 *
d>1.40 amp_aff: 0.00000686 +/- 0.00000221

---- fit on data from 1.50 to 50.00 cM (unconstrained affine term) ----
d>1.50 decay: 13.26 +/- 1.55 z = 8.55 *
d>1.50 amp_tot: 0.00012924 +/- 0.00000953
d>1.50 amp_exp: 0.00012597 +/- 0.00000927 z = 13.58 *
d>1.50 amp_aff: 0.00000653 +/- 0.00000223

---- fit on data from 1.60 to 50.00 cM (unconstrained affine term) ----
d>1.60 decay: 13.00 +/- 1.53 z = 8.50 *
d>1.60 amp_tot: 0.00012727 +/- 0.00000970
d>1.60 amp_exp: 0.00012409 +/- 0.00000941 z = 13.19 *
d>1.60 amp_aff: 0.00000637 +/- 0.00000226

<u>ALDER linkage disequilibrium decay rates for Anatolia_Neolithic and Iberia_HG:</u>

---- fit on data from 1.00 to 50.00 cM (unconstrained affine term) ----
d>1.00 decay: 18.06 +/- 4.15 z = 4.35 *
d>1.00 amp_tot: 0.00016610 +/- 0.00002088
d>1.00 amp_exp: 0.00016126 +/- 0.00001984 z = 8.13 *
d>1.00 amp_aff: 0.00000968 +/- 0.00000372

---- fit on data from 1.10 to 50.00 cM (unconstrained affine term) ----
d>1.10 decay: 17.66 +/- 4.37 z = 4.05 *
d>1.10 amp_tot: 0.00016305 +/- 0.00002265
d>1.10 amp_exp: 0.00015829 +/- 0.00002155 z = 7.35 *
d>1.10 amp_aff: 0.00000952 +/- 0.00000381

---- fit on data from 1.20 to 50.00 cM (unconstrained affine term) ----
d>1.20 decay: 17.49 +/- 4.44 z = 3.94 *
d>1.20 amp_tot: 0.00016171 +/- 0.00002342
d>1.20 amp_exp: 0.00015699 +/- 0.00002226 z = 7.05 *
d>1.20 amp_aff: 0.00000945 +/- 0.00000388

---- fit on data from 1.30 to 50.00 cM (unconstrained affine term) ----
d>1.30 decay: 17.12 +/- 4.58 z = 3.74 *
d>1.30 amp_tot: 0.00015881 +/- 0.00002412
d>1.30 amp_exp: 0.00015416 +/- 0.00002271 z = 6.79 *
d>1.30 amp_aff: 0.00000930 +/- 0.00000407

---- fit on data from 1.40 to 50.00 cM (unconstrained affine term) ----
d>1.40 decay: 16.92 +/- 4.66 z = 3.63 *
d>1.40 amp_tot: 0.00015717 +/- 0.00002500
d>1.40 amp_exp: 0.00015257 +/- 0.00002350 z = 6.49 *
d>1.40 amp_aff: 0.00000921 +/- 0.00000417

<u>ALDER linkage disequilibrium decay rates for Anatolia_Neolithic and WHG:</u>

---- fit on data from 1.00 to 50.00 cM (unconstrained affine term) ----
d>1.00 decay: 16.92 +/- 1.64 z = 10.30 *
d>1.00 amp_tot: 0.00019322 +/- 0.00001625
d>1.00 amp_exp: 0.00018754 +/- 0.00001669 z = 11.23 *

d>1.00 amp_aff: 0.00001136 +/- 0.00000259

---- fit on data from 1.10 to 50.00 cM (unconstrained affine term) ----
d>1.10 decay: 16.08 +/- 1.45 z = 11.05 *
d>1.10 amp_tot: 0.00018517 +/- 0.00001579
d>1.10 amp_exp: 0.00017972 +/- 0.00001625 z = 11.06 *
d>1.10 amp_aff: 0.00001090 +/- 0.00000260

---- fit on data from 1.20 to 50.00 cM (unconstrained affine term) ----
d>1.20 decay: 15.39 +/- 1.37 z = 11.24 *
d>1.20 amp_tot: 0.00017843 +/- 0.00001540
d>1.20 amp_exp: 0.00017318 +/- 0.00001584 z = 10.94 *
d>1.20 amp_aff: 0.00001050 +/- 0.00000263

---- fit on data from 1.30 to 50.00 cM (unconstrained affine term) ----
d>1.30 decay: 14.94 +/- 1.36 z = 10.96 *
d>1.30 amp_tot: 0.00017394 +/- 0.00001537
d>1.30 amp_exp: 0.00016883 +/- 0.00001580 z = 10.69 *
d>1.30 amp_aff: 0.00001023 +/- 0.00000263

---- fit on data from 1.40 to 50.00 cM (unconstrained affine term) ----
d>1.40 decay: 14.98 +/- 1.37 z = 10.92 *
d>1.40 amp_tot: 0.00017433 +/- 0.00001528
d>1.40 amp_exp: 0.00016920 +/- 0.00001572 z = 10.76 *
d>1.40 amp_aff: 0.00001026 +/- 0.00000263

ALDER linkage disequilibrium decay rates for Anatolia_Neolithic and Loschbour HG:

---- fit on data from 1.00 to 50.00 cM (unconstrained affine term) ----
d>1.00 decay: 14.85 +/- 2.82 z = 5.28 *
d>1.00 amp_tot: 0.00021335 +/- 0.00002318
d>1.00 amp_exp: 0.00020647 +/- 0.00002264 z = 9.12 *
d>1.00 amp_aff: 0.00001376 +/- 0.00000548

---- fit on data from 1.10 to 50.00 cM (unconstrained affine term) ----
d>1.10 decay: 14.25 +/- 2.74 z = 5.21 *
d>1.10 amp_tot: 0.00020650 +/- 0.00002296
d>1.10 amp_exp: 0.00019986 +/- 0.00002236 z = 8.94 *
d>1.10 amp_aff: 0.00001327 +/- 0.00000558

---- fit on data from 1.20 to 50.00 cM (unconstrained affine term) ----
d>1.20 decay: 13.68 +/- 2.53 z = 5.40 *
d>1.20 amp_tot: 0.00019987 +/- 0.00002147
d>1.20 amp_exp: 0.00019348 +/- 0.00002095 z = 9.24 *
d>1.20 amp_aff: 0.00001276 +/- 0.00000561

---- fit on data from 1.30 to 50.00 cM (unconstrained affine term) ----
d>1.30 decay: 13.25 +/- 2.47 z = 5.36 *
d>1.30 amp_tot: 0.00019480 +/- 0.00002137
d>1.30 amp_exp: 0.00018861 +/- 0.00002084 z = 9.05 *
d>1.30 amp_aff: 0.00001237 +/- 0.00000567

---- fit on data from 1.40 to 50.00 cM (unconstrained affine term) ----
d>1.40 decay: 13.19 +/- 2.51 z = 5.26 *
d>1.40 amp_tot: 0.00019405 +/- 0.00002266
d>1.40 amp_exp: 0.00018789 +/- 0.00002210 z = 8.50 *
d>1.40 amp_aff: 0.00001231 +/- 0.00000569

DATES results for admixture between Anatolian farmers and the following HG populations:

Pop	generations	stedrr
OrienteC_HG	51,977	19,997
Croatia_Mesolithic_HG	30,954	8,068
Bichon	20,749	4,27
Blatterhohle_MN	18,001	3,072
Koros_Hungary_EN_HG	28,386	6,018
Serbia_HG	22,412	3,977
Serbia_Mesolithic_Neolithic	20,339	2,842
Narva_LT	17,153	2,482
Iron_Gates_HG	18,416	1,967
Loschbour	16,608	2,654
Iberia_HG	22,208	3,853
Latvia_EN	27,054	7,858
Baalberge_MN	9,95	55,816
France_MN	94,043	19,491
Latvia_HG	18,359	2,001

Supplementary Table 1: AMS and EA-IRMS results from dated bone samples. KIA-53048 is the weighted mean of two graphite targets, which gave consistent ^{14}C ages (4412±24, 4424±28). EA-IRMS data are average results from four measurements of each sample, with standard deviations <0.1 ‰ for both isotopes.

sample	element	collagen yield %	AMS lab number	^{14}C age (BP)	atomic C/N	$\delta^{13}C$	$\delta^{15}N$
KH150203	cranium	9.7	KIA-52267	4481±24	3.2	-20.5	10.9
KH150287	cranium	1.9	KIA-52268	4499±25	3.2	-20.6	11.4
KH150288	cranium	7.0	KIA-52269	4512±26	3.2	-20.6	10.5
KH150289	cranium	9.5	KIA-52270	4499±24	3.2	-20.7	11.0
KH150422	tooth	12.0	KIA-52272	4538±24	3.2	-20.4	10.4
KH150610	petrous	11.4	KIA-52273	4532±25	3.2	-19.7	12.6
KH150611	petrous	3.8	KIA-53044	4518±25	3.2	-20.7	11.1
KH150613	petrous	5.7	KIA-53045	4499±26	3.2	-22.0	11.6
KH150615	petrous	6.4	KIA-53046	4462±24	3.1	-20.8	10.7
KH150616	petrous	5.1	KIA-52274	4492±24	3.2	-21.3	11.1
KH150618	petrous	4.2	KIA-52275	4468±25	3.2	-20.5	11.4
KH150619	petrous	9.1	KIA-52276	4455±24	3.2	-20.7	9.7
KH150620	petrous	5.1	KIA-53047	4491±25	3.2	-19.7	13.1
KH150622	petrous	8.4	KIA-53048	4417±19	3.1	-20.4	14.1
KH150625	petrous	5.0	KIA-52277	4497±27	3.2	-19.8	13.0
KH150627	petrous	2.8	KIA-52278	4462±27	3.2	-21.1	10.7
KH150628	petrous	9.9	KIA-53049	4448±26	3.1	-20.3	11.5
KH150630	petrous	5.6	KIA-53050	4564±25	3.2	-20.4	10.3
KH150633	petrous	7.0	KIA-52279	4486±29	3.2	-20.3	9.9
KH150634	petrous	8.8	KIA-53051	4507±25	3.2	-20.4	10.9
KH150635	petrous	9.6	KIA-52280	4425±28	3.2	-20.6	12.8
KH150636	petrous	4.6	KIA-53052	4493±26	3.2	-20.4	10.8
KH150637	petrous	4.7	KIA-52281	4432±28	3.2	-20.6	11.9
KH150640	petrous	6.4	KIA-53053	4461±25	3.2	-20.8	11.6
KH150641	petrous	7.5	KIA-52282	4473±28	3.2	-20.4	12.2

Supplementary Table 2: Viruses included in the HLA peptide binding prediction analysis reported together with their proteome identifier (UPID) from UniProt databases.

Viruses	Proteome identifier (UPID)
Epstein-Barr virus	UP000153037
Hepatitis B virus	UP000007930
Hepatitis C virus	UP000000518
Human immunodeficiency virus	UP000002241
Human herpesvirus	UP000006930
Influenza A virus	UP000009255
Measles virus	UP000008699
Mumps virus	UP000002331
Rabies virus	UP000008649
Rubella virus	UP000000571
Variola virus	UP000002060

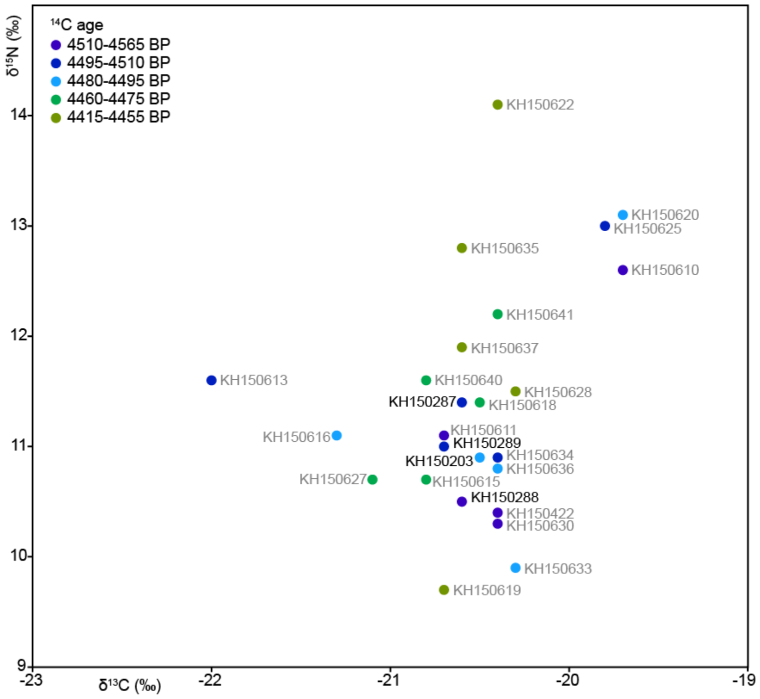

Supplementary Figure 1: Dietary stable isotopes from the dated samples (Supplementary Table 1). Colors denote the ^{14}C ages obtained on the same collagen extracts. Black labels: cranial bone; grey labels: petrous bone, or dentine (KH150422).

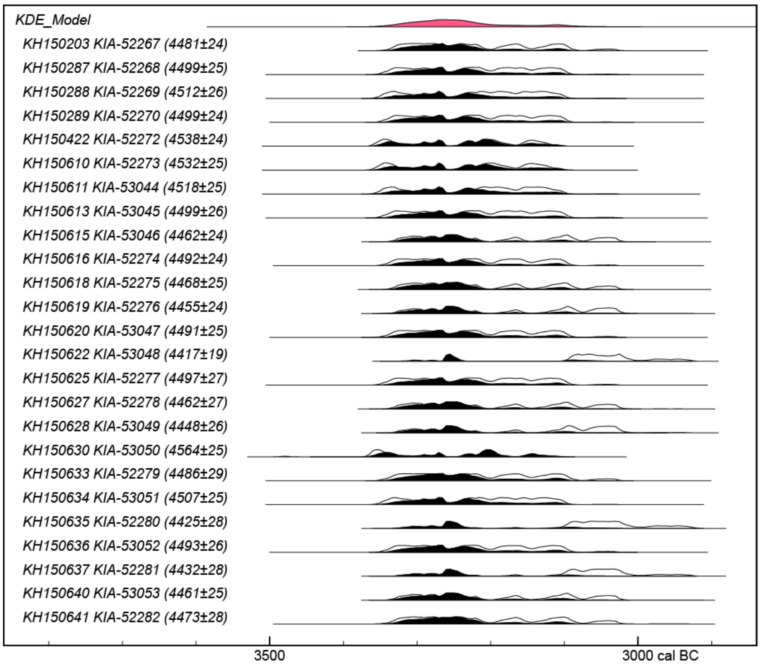

Supplementary Figure 2: Calibrated individual ^{14}C ages (9, 10) (outline distributions) and posterior density estimates for the calendar dates of these samples (solid distributions) obtained using the OxCal function KDE_Model (11). The pink distribution *KDE_Model* summarizes the results.

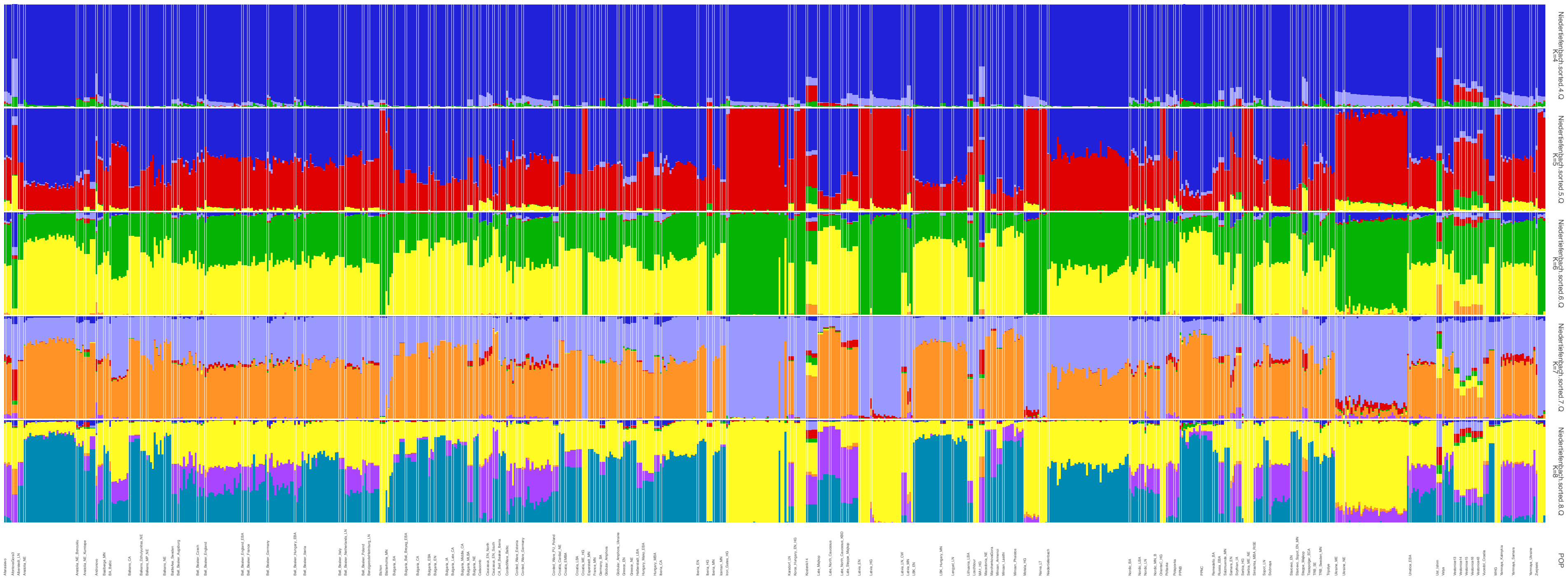

Supplementary Figure 3: Unsupervised admixture analysis for K=4, 5, 6, 7 and 8 ancestral genetic components for 123 selected ancient populations/individuals. Modern-day populations were included in the analysis but are not shown. BA=Bronze Age, EBA=Early Bronze Age, EN=Eneolithic, HG=hunter-gatherer, IA=Iron Age, LBA=Late Bronze Age, LBK=Linearbandkeramik, LN=Late Neolithic, M(L)BA=Middle (Late) Bronze Age, MN=Middle Neolithic, PPNA=Pre-Pottery Neolithic A, PPNB=Pre-Pottery Neolithic B, PU=Proto-Unetice, TRB=Trichterbecher (Funnel Beaker Culture, FBC).

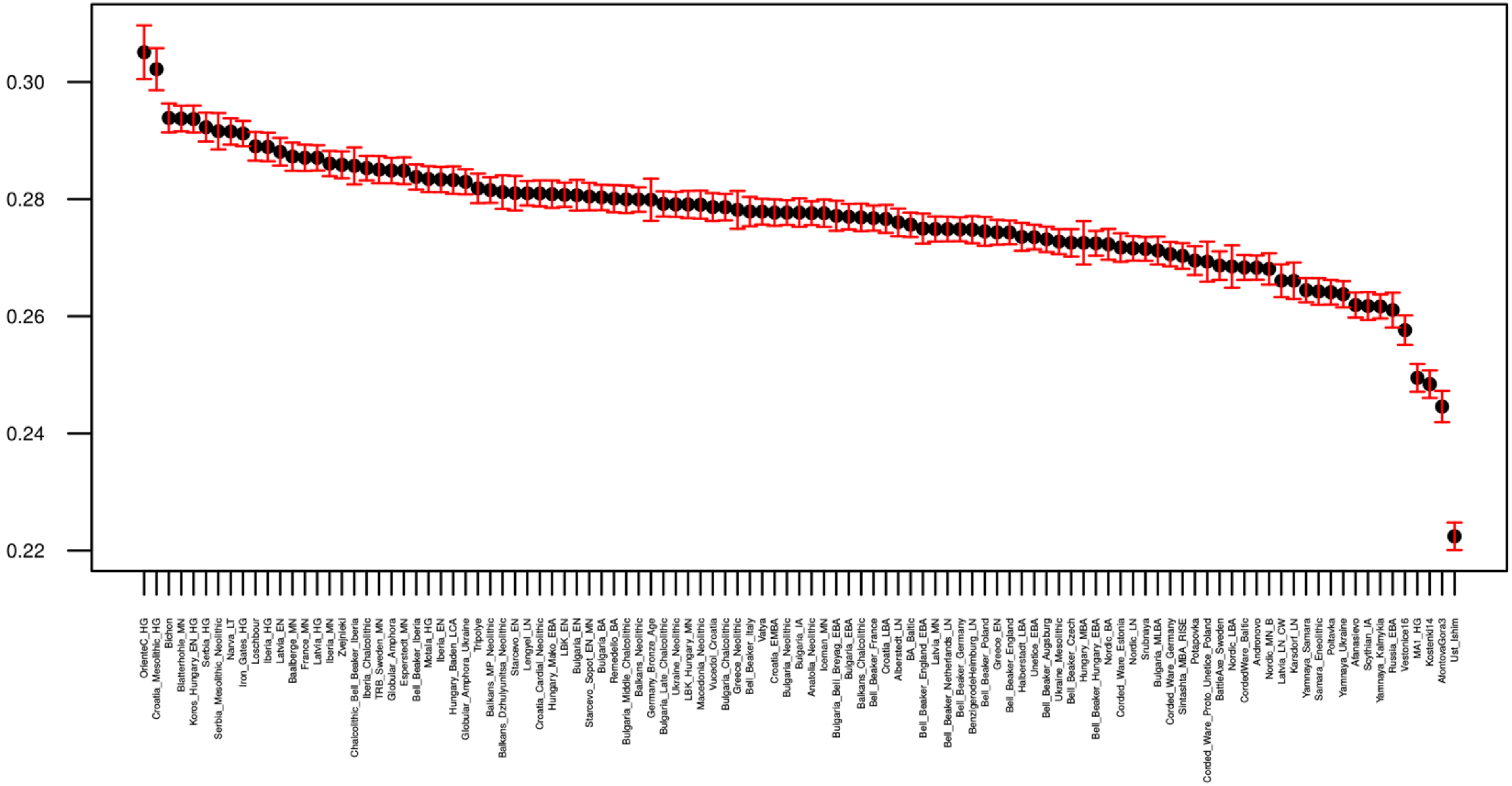

Supplementary Figure 4: f3-outgroup statistics *f3(Niedertiefenbach; Test, Mbuti)* showing the amount of shared genetic drift between Niedertiefenbach and previously published ancient populations/individuals. EBA=Early Bronze Age, EN=Eneolithic, HG=hunter-gatherer, IA=Iron Age, LBA=Late Bronze Age, LBK=Linearbandkeramik, LN=Late Neolithic, M(L)BA=Middle (Late) Bronze Age, MN=Middle Neolithic, PU=Proto-Unetice, TRB=Trichterbecher (Funnel Beaker Culture, FBC).

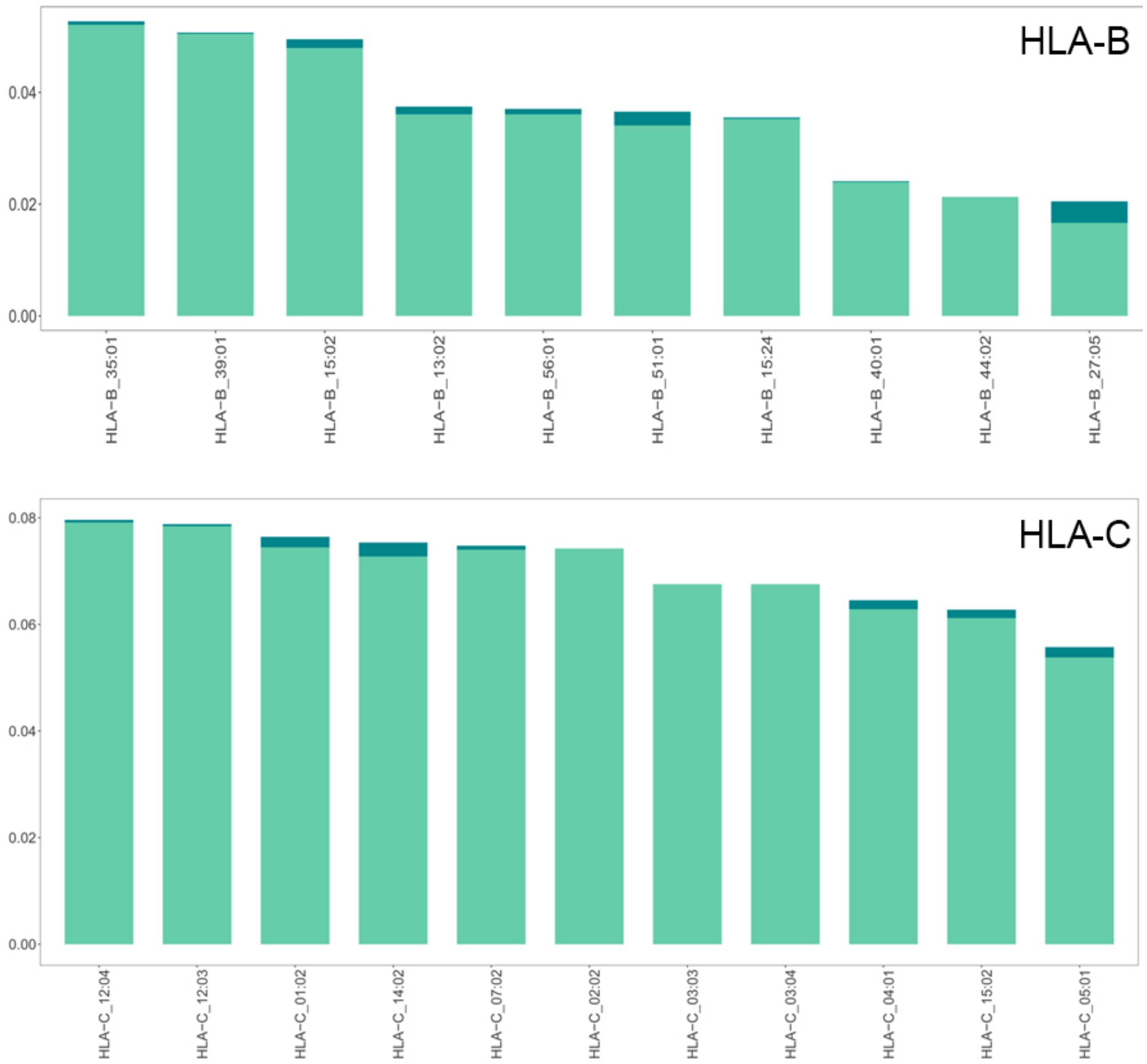

Supplementary Figure 5: Computationally predicted fraction of viral peptides (N = 164,037) bound by the HLA-B and HLA-C alleles observed in the Niedertiefenbach individuals. Total fraction (light green) as well as unique fraction (peptides uniquely bound by this allele, dark green) are shown. For estimation of unique binding, all 58 HLA-B and 29 HLA-C alleles observed in this study (including both ancient and modern samples) were analyzed. See Supplementary Table 2 for list of included viruses.

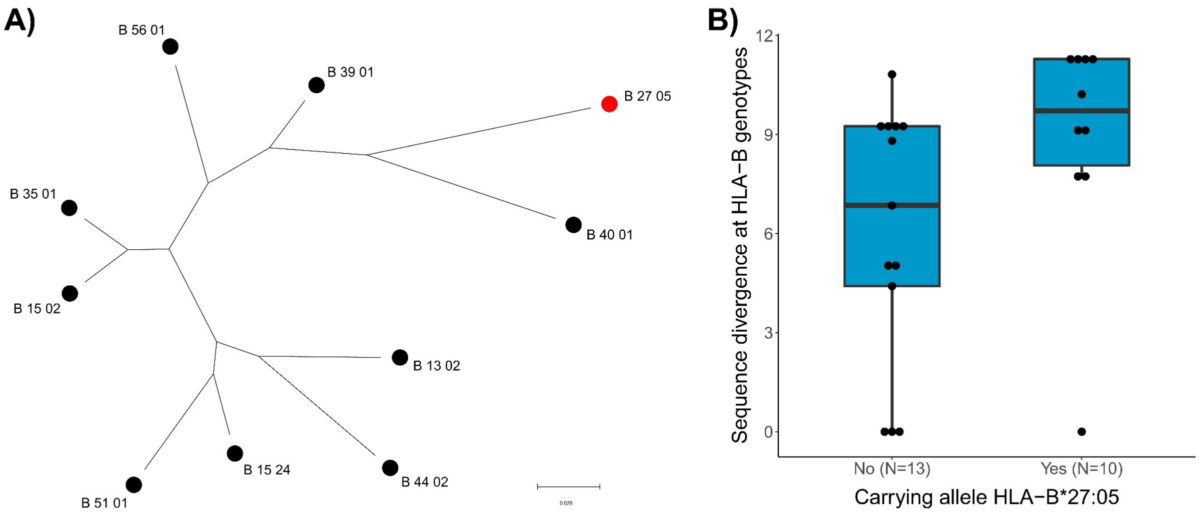

Supplementary Figure 6: Amino acid sequence divergence at HLA-B. **A)** Phylogenetic relationship of the 10 HLA-B alleles observed among Niedertiefenbach individuals. Maximum-likelihood tree based on amino acid sequence (HLA-B*27:05 marked in red). **B)** Comparison of HLA-B amino acid allele divergence between Niedertiefenbach individuals with and without the allele HLA-B*27:05 (MWUtest, *P* = 0.061)

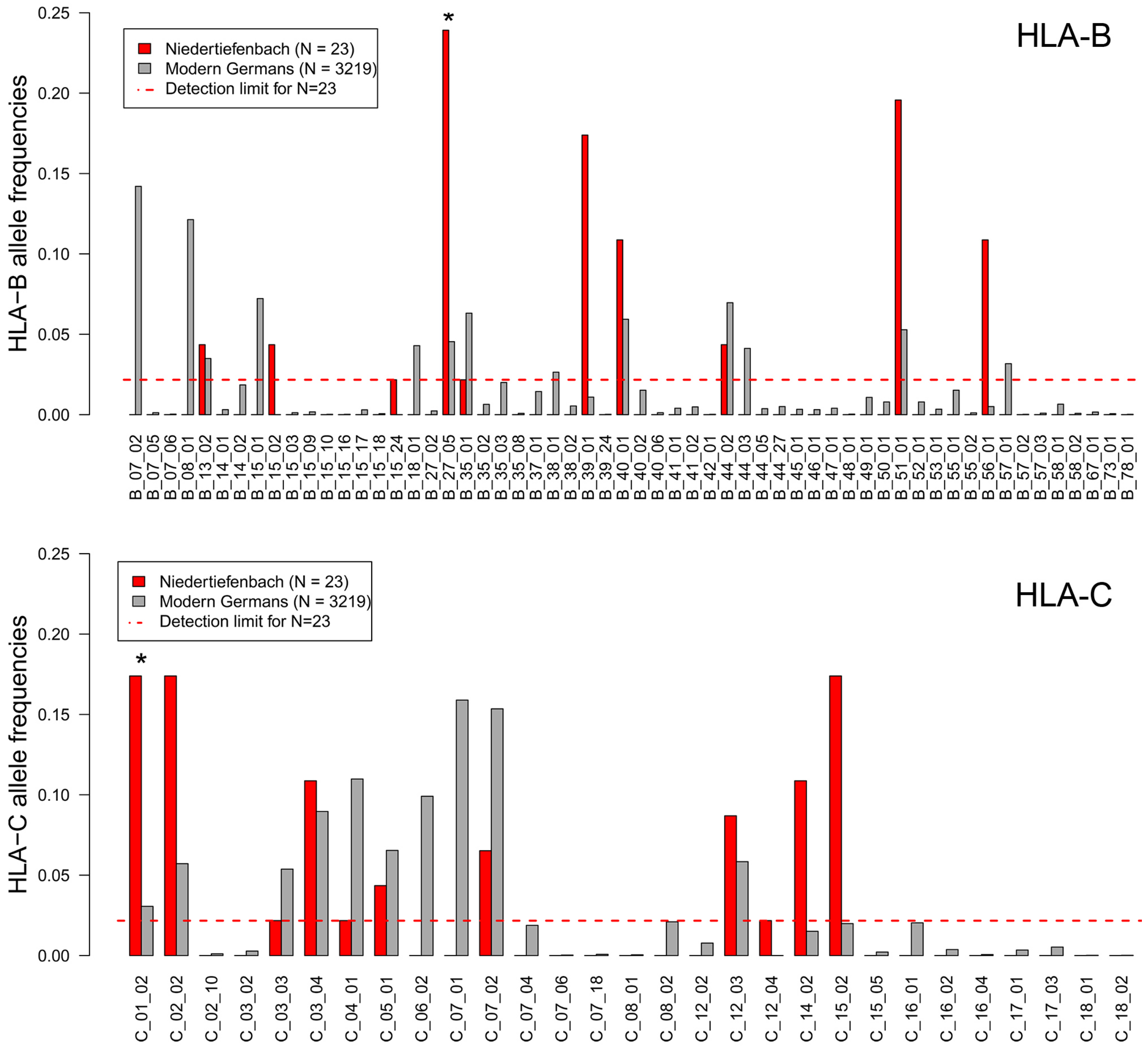

Supplementary Figure 7: Allele frequencies in the Niedertiefenbach individuals (N = 23, red) and a modern German cohort of healthy individuals (N = 3,219) for HLA-B and HLA-C. The dashed red line indicates the detection limit for rare alleles with a sample size of N = 23.

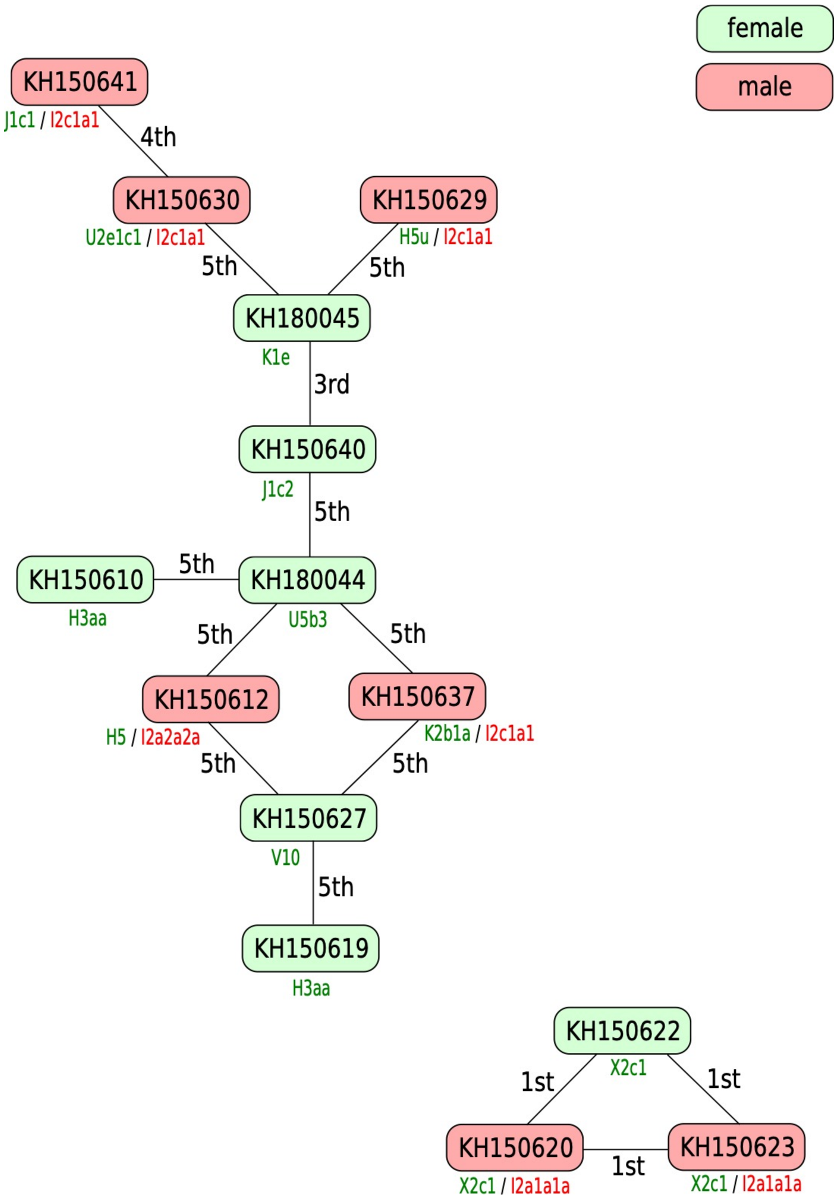

Supplementary Figure 8: Kinship analysis estimating the degree of relatedness among the 42 Niedertiefenbach individuals. Those individuals not shown are unrelated to any of the others. Mitochondrial haplotypes are depicted in green, Y haplotypes in red.

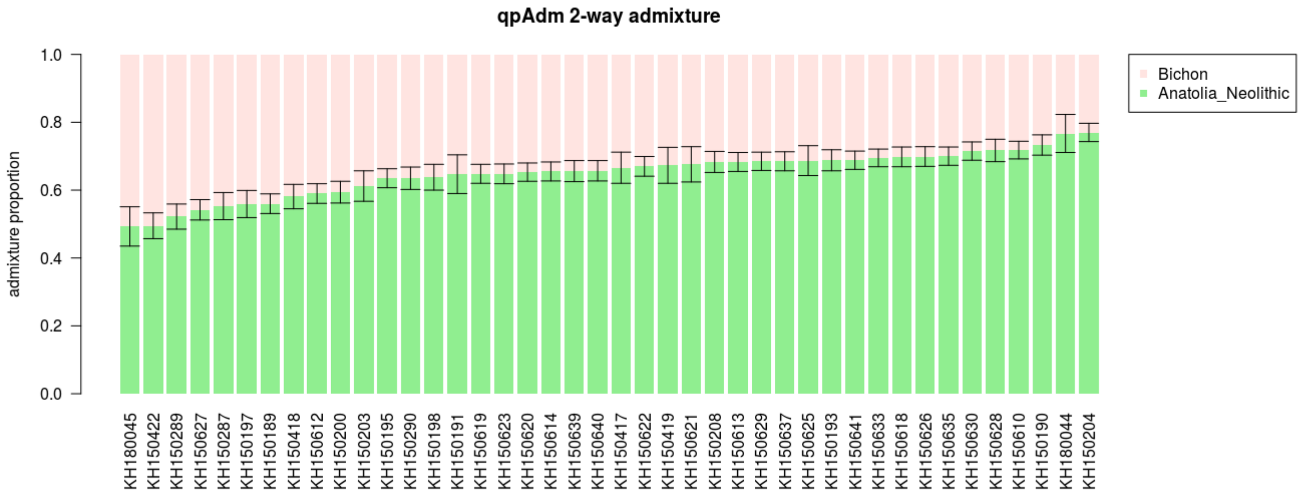
qpAdm 2-way admixture
admixture proportion
1.0
0.8
0.6
0.4
0.2
0.0
Bichon
Anatolia_Neolithic
KH180045 KH150422 KH150289 KH150627 KH150287 KH150197 KH150189 KH150418 KH150612 KH150200 KH150203 KH150195 KH150290 KH150198 KH150191 KH150619 KH150623 KH150620 KH150614 KH150639 KH150640 KH150417 KH150622 KH150419 KH150621 KH150208 KH150613 KH150629 KH150637 KH150625 KH150193 KH150641 KH150633 KH150618 KH150626 KH150635 KH150630 KH150628 KH150610 KH150190 KH180044 KH150204

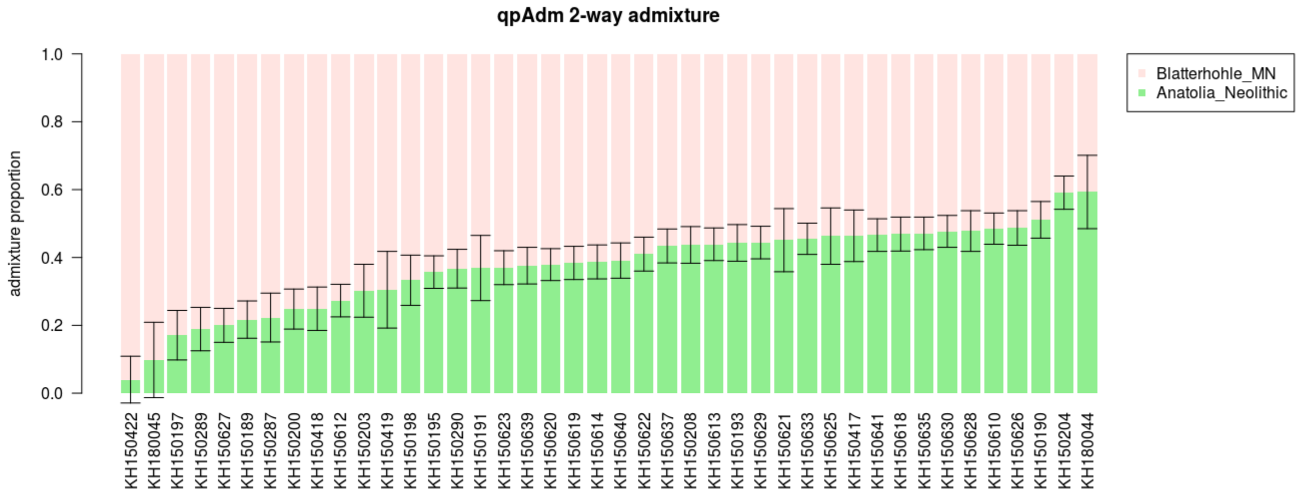
qpAdm 2-way admixture
admixture proportion
1.0
0.8
0.6
0.4
0.2
0.0
Blatterhohle_MN
Anatolia_Neolithic
KH150422 KH180045 KH150197 KH150289 KH150627 KH150189 KH150287 KH150200 KH150418 KH150612 KH150203 KH150419 KH150198 KH150195 KH150290 KH150191 KH150623 KH150639 KH150620 KH150619 KH150614 KH150640 KH150622 KH150637 KH150208 KH150613 KH150193 KH150629 KH150621 KH150633 KH150625 KH150417 KH150641 KH150618 KH150635 KH150630 KH150628 KH150610 KH150626 KH150190 KH150204 KH180044

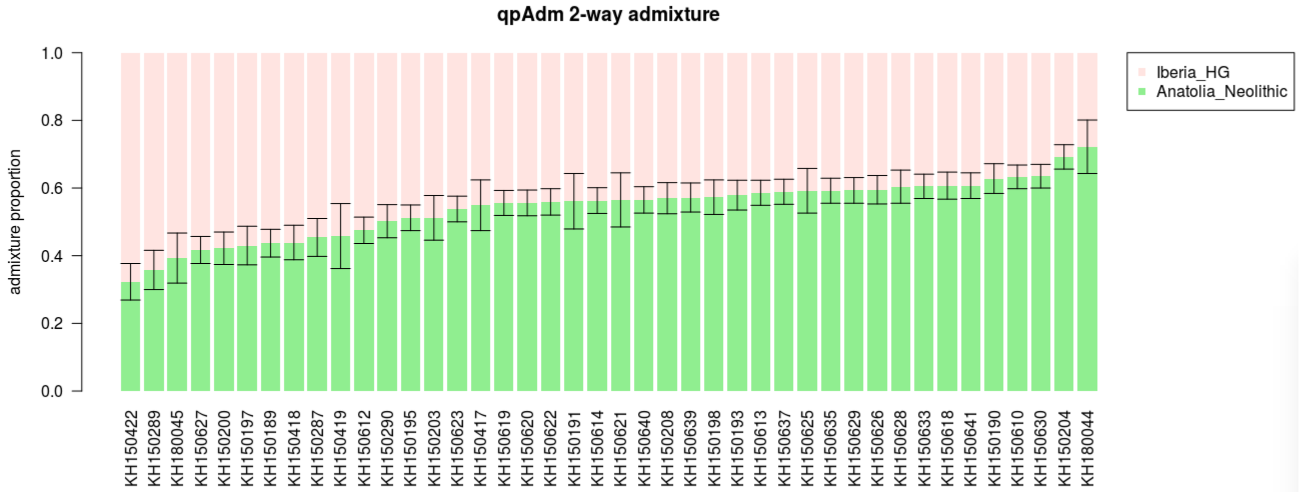
qpAdm 2-way admixture
admixture proportion
1.0
0.8
0.6
0.4
0.2
0.0
Iberia_HG
Anatolia_Neolithic
KH150422 KH150289 KH180045 KH150627 KH150200 KH150197 KH150189 KH150418 KH150287 KH150419 KH150612 KH150290 KH150195 KH150203 KH150623 KH150417 KH150619 KH150620 KH150622 KH150191 KH150614 KH150621 KH150640 KH150208 KH150639 KH150198 KH150193 KH150613 KH150637 KH150625 KH150635 KH150629 KH150626 KH150628 KH150633 KH150618 KH150641 KH150190 KH150610 KH150630 KH150204 KH180044

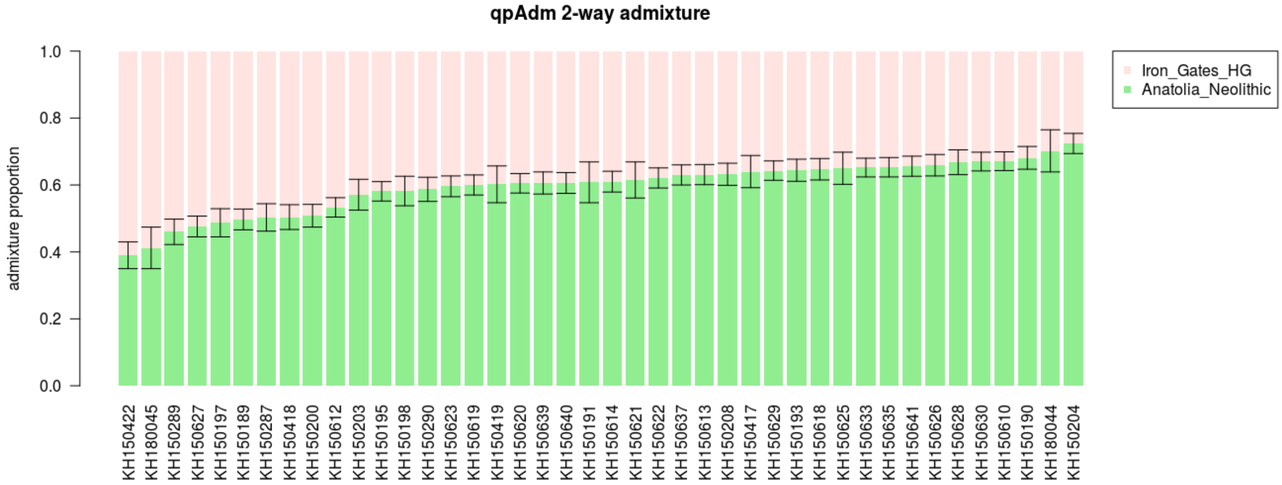
qpAdm 2-way admixture
admixture proportion
1.0
0.8
0.6
0.4
0.2
0.0
Iron_Gates_HG
Anatolia_Neolithic
KH150422
KH180045
KH150289
KH150627
KH150197
KH150189
KH150287
KH150418
KH150200
KH150612
KH150203
KH150195
KH150198
KH150290
KH150623
KH150619
KH150419
KH150620
KH150639
KH150640
KH150191
KH150614
KH150621
KH150622
KH150637
KH150613
KH150208
KH150417
KH150629
KH150193
KH150618
KH150625
KH150633
KH150635
KH150641
KH150626
KH150628
KH150630
KH150610
KH150190
KH180044
KH150204

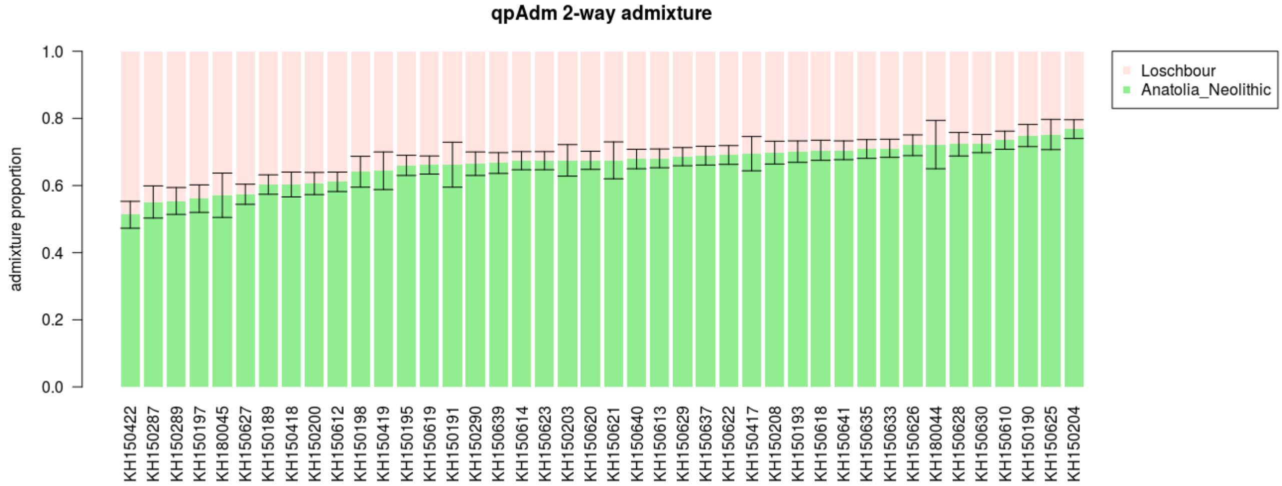
qpAdm 2-way admixture
admixture proportion
1.0
0.8
0.6
0.4
0.2
0.0
Loschbour
Anatolia_Neolithic
KH150422
KH150287
KH150289
KH150197
KH180045
KH150627
KH150189
KH150418
KH150200
KH150612
KH150198
KH150419
KH150195
KH150619
KH150191
KH150290
KH150639
KH150614
KH150623
KH150203
KH150620
KH150621
KH150640
KH150613
KH150629
KH150637
KH150622
KH150417
KH150208
KH150193
KH150618
KH150641
KH150635
KH150633
KH150626
KH180044
KH150628
KH150630
KH150610
KH150190
KH150625
KH150204

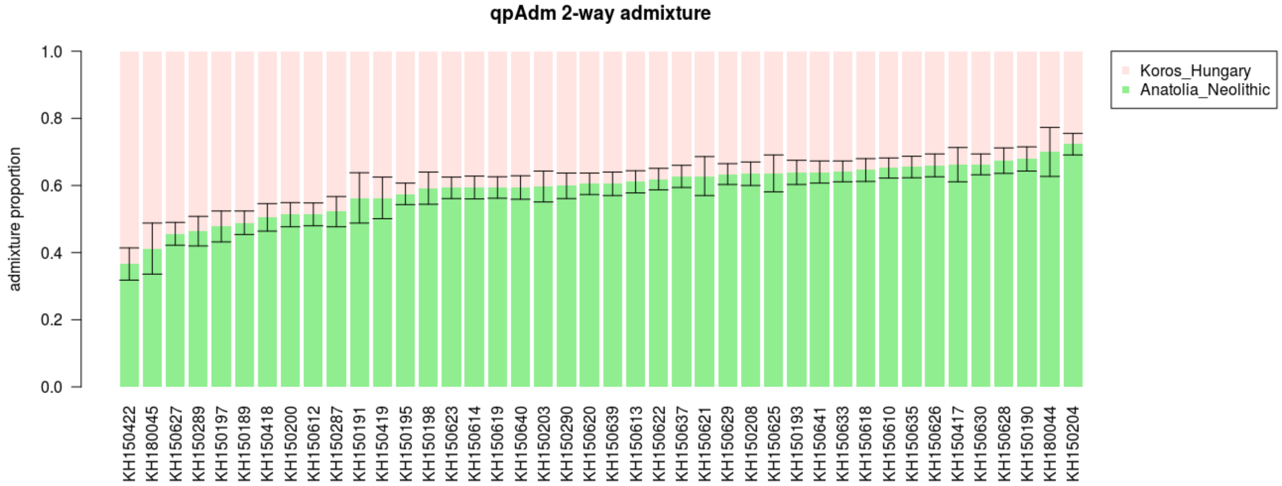
qpAdm 2-way admixture
admixture proportion
1.0
0.8
0.6
0.4
0.2
0.0
Koros_Hungary
Anatolia_Neolithic
KH150422
KH180045
KH150627
KH150289
KH150197
KH150189
KH150418
KH150200
KH150612
KH150287
KH150191
KH150419
KH150195
KH150198
KH150623
KH150614
KH150619
KH150640
KH150203
KH150290
KH150620
KH150639
KH150613
KH150622
KH150637
KH150621
KH150629
KH150208
KH150625
KH150193
KH150641
KH150633
KH150618
KH150610
KH150635
KH150626
KH150417
KH150630
KH150628
KH150190
KH180044
KH150204

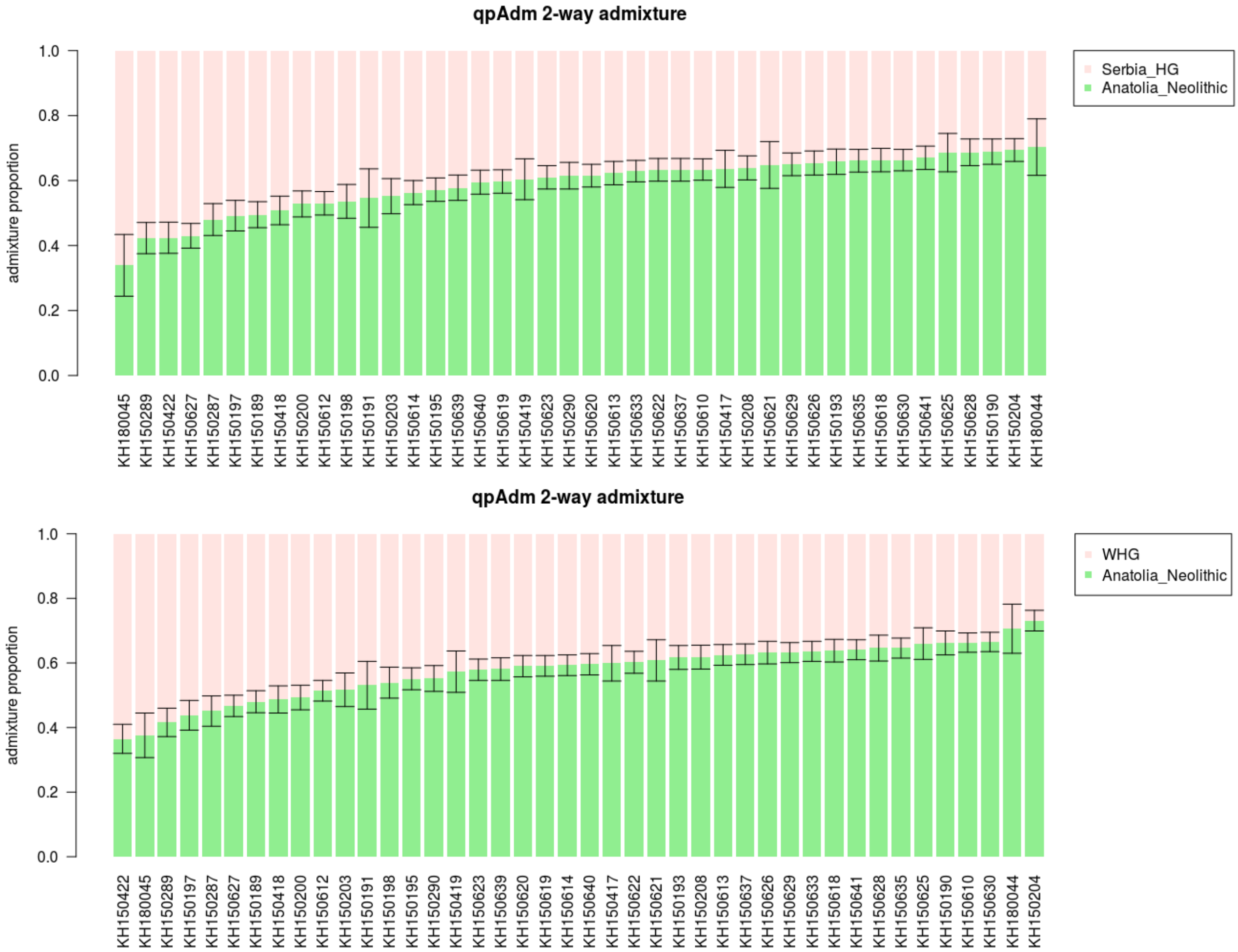

Supplementary Figure 9: Results of the qpAdm 2-way models for each Niedertiefenbach individual (x-axis). Bar plots show the estimated admixture proportions (y-axis) of Anatolia_Neolithic (green) and different HG populations (pink). Bars indicate standard errors provided by qpAdm.

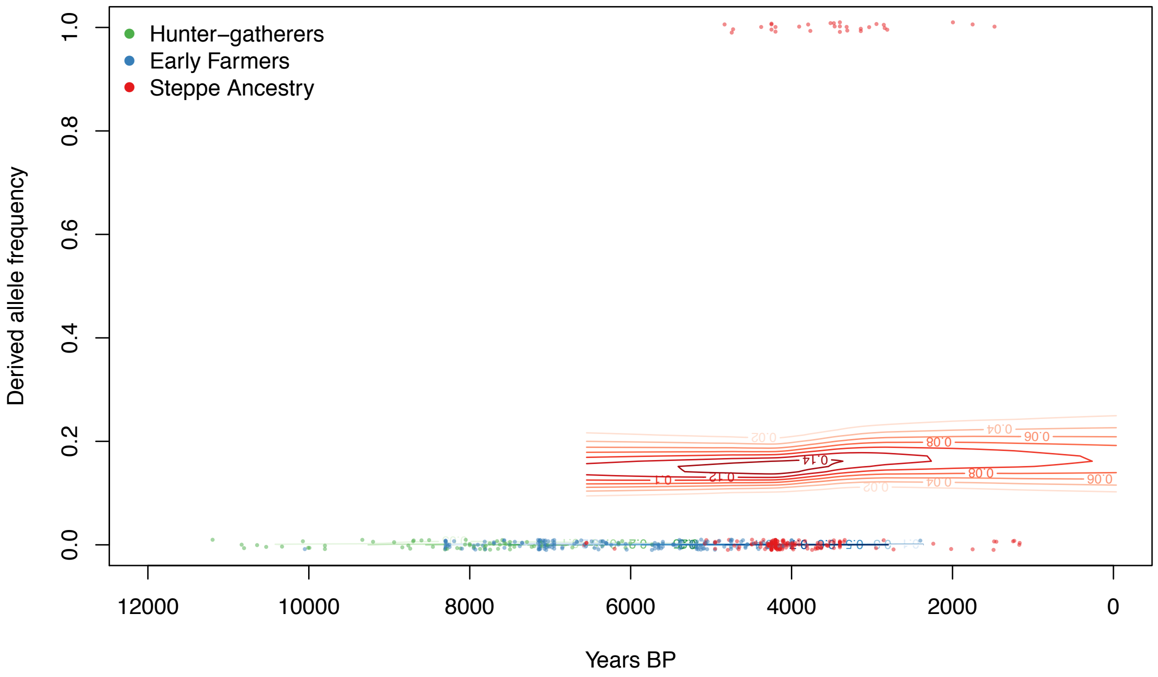

Supplementary Figure 10: Frequency of the rs3135388 T allele that is associated with HLA-DRB1*15:01. Frequency calculations are based on 140 hunter-gatherers, 455 early farmers and 460 individuals with steppe population-related ancestry.

References

1. Longin, R. New method of collagen extraction for radiocarbon dating. *Nature* **230**: 241-242 (1971).

2. Nadeau M-J. *et al.* Sample throughput and data quality at the Leibniz-Labor AMS facility. *Radiocarbon* **40**: 239-245 (1998).

3. Grootes, PM., Nadeau, M-J. & Rieck, A. ^{14}C-AMS at the Leibniz-Labor: radiometric dating and isotope research. *Nuclear Instruments and Methods in Physics Research Section B: Beam Interactions with Materials and Atoms* **223**: 55-61 (2004).

4. Sieper, H-P. *et al.* A measuring system for the fast simultaneous isotope ratio and elemental analysis of carbon, hydrogen, nitrogen and sulfur in food commodities and other biological material. *Rapid Commun Mass Sp.* **20:** 2521-2527 (2006).

5. Szpak, P. Fish bone chemistry and ultrastructure: implications for taphonomy and stable isotope analysis. *J Archaeol Sci* **38**: 3358-3372 (2011).

6. Ward, GK. & Wilson, SR. Procedures for comparing and combining radiocarbon age-determinations - a critique. *Archaeometry* **20**: 19-31 (1978).

7. Fernandes, R., Grootes, P., Nadeau, M-J. & Nehlich, O. Quantitative diet reconstruction of a Neolithic population using a Bayesian mixing model (FRUITS): The case study of Ostorf (Germany). *Am J Phys Anthropol* **158**: 325-340 (2015).

8. Jørkov, MLS., Heinemeier, J. & Lynnerup, N. The petrous bone—A new sampling site for identifying early dietary patterns in stable isotopic studies. *Am J Phys Anthropol* **138**: 199-209 (2009).

9. Ramsey, CB. Bayesian analysis of radiocarbon dates. *Radiocarbon* **51**: 337-360 (2009).

10. Reimer, PJ. et al. IntCal13 and Marine13 radiocarbon age calibration curves 0-50,000 years cal BP. *Radiocarbon* **55**: 1869-1887 (2013).

11. Ramsey, CB. Methods for summarizing radiocarbon datasets. *Radiocarbon* **59**: 1809-1833 (2017).
