# Supplementary material for: Genome-wide study of a Neolithic Wartberg grave community reveals distinct HLA variation and hunter-gatherer ancestry
Source: Commun Biol. 2021 Jan 25;4:113. doi: 10.1038/s42003-020-01627-4 (PMC7835224; doi:10.1038/s42003-020-01627-4)
Supplement: Supplementary file 3 — Description of Additional Supplementary Files [file 42003_2020_1627_MOESM3_ESM.pdf]

Description of Additional Supplementary Files

File name: Supplementary Data 1

Description: Overview of samples

File name: Supplementary Data 2

Description: qpADM models for Niedertiefenbach

File name: Supplementary Data 3

Description: Overview of HLA class I and II calls
