# Supplementary material for: Genome-wide study of a Neolithic Wartberg grave community reveals distinct HLA variation and hunter-gatherer ancestry
Source: Commun Biol. 2021 Jan 25;4:113. doi: 10.1038/s42003-020-01627-4 (PMC7835224; doi:10.1038/s42003-020-01627-4)
Supplement: Supplementary file 8 — Reporting Summary [file 42003_2020_1627_MOESM8_ESM.pdf]

nature research

Corresponding author(s): Ben Krause-Kyora

Last updated by author(s): Dec 17, 2020

Reporting Summary

Nature Research wishes to improve the reproducibility of the work that we publish. This form provides structure for consistency and transparency in reporting. For further information on Nature Research policies, see our Editorial Policies and the Editorial Policy Checklist.

Statistics

For all statistical analyses, confirm that the following items are present in the figure legend, table legend, main text, or Methods section.

n/a	Confirmed	
☐	☒	The exact sample size (n) for each experimental group/condition, given as a discrete number and unit of measurement
☒	☐	A statement on whether measurements were taken from distinct samples or whether the same sample was measured repeatedly
☐	☒	The statistical test(s) used AND whether they are one- or two-sided<br>*Only common tests should be described solely by name; describe more complex techniques in the Methods section.*
☒	☐	A description of all covariates tested
☒	☐	A description of any assumptions or corrections, such as tests of normality and adjustment for multiple comparisons
☐	☒	A full description of the statistical parameters including central tendency (e.g. means) or other basic estimates (e.g. regression coefficient) AND variation (e.g. standard deviation) or associated estimates of uncertainty (e.g. confidence intervals)
☒	☐	For null hypothesis testing, the test statistic (e.g. F, t, r) with confidence intervals, effect sizes, degrees of freedom and P value noted<br>*Give P values as exact values whenever suitable.*
☐	☒	For Bayesian analysis, information on the choice of priors and Markov chain Monte Carlo settings
☒	☐	For hierarchical and complex designs, identification of the appropriate level for tests and full reporting of outcomes
☐	☒	Estimates of effect sizes (e.g. Cohen's d, Pearson's r), indicating how they were calculated

Our web collection on statistics for biologists contains articles on many of the points above.

Software and code

Policy information about availability of computer code

Data collection	A description of the data collection can be found in the material and methods section.
Data analysis	A description of the data analysis can be found in the material and methods section.

For manuscripts utilizing custom algorithms or software that are central to the research but not yet described in published literature, software must be made available to editors and reviewers. We strongly encourage code deposition in a community repository (e.g. GitHub). See the Nature Research guidelines for submitting code & software for further information.

Data

Policy information about availability of data

All manuscripts must include a data availability statement. This statement should provide the following information, where applicable:

- Accession codes, unique identifiers, or web links for publicly available datasets
- A list of figures that have associated raw data
- A description of any restrictions on data availability

The aligned sequences are available through the European Nucleotide Archive under accession number ERP118364. Analyzed skeleton material belonging to the Landesamt für Denkmalpflege Hessen, hessenARCHÄOLOGIE.

Field-specific reporting

Life sciences study design

All studies must disclose on these points even when the disclosure is negative.

Sample size	We sampled 89 randomly selected individuals out of a total of about 150 excavated individuals.
Data exclusions	Data that did not contain human DNA or showed contamination were excluded.
Replication	no replication
Randomization	All samples belong to one group
Blinding	N/A

Reporting for specific materials, systems and methods

We require information from authors about some types of materials, experimental systems and methods used in many studies. Here, indicate whether each material, system or method listed is relevant to your study. If you are not sure if a list item applies to your research, read the appropriate section before selecting a response.

Materials & experimental systems

n/a	Involved in the study
☒	☐ Antibodies
☒	☐ Eukaryotic cell lines
☐	☒ Palaeontology and archaeology
☒	☐ Animals and other organisms
☒	☐ Human research participants
☒	☐ Clinical data
☒	☐ Dual use research of concern

Methods

n/a	Involved in the study
☒	☐ ChIP-seq
☒	☐ Flow cytometry
☒	☐ MRI-based neuroimaging

Palaeontology and Archaeology

Specimen provenance	The samples are the property of the Landesamt für Denkmalpflege Hessen, hessenARCHÄOLOGIE. Permission was granted by the Landesamt für Denkmalpflege Hessen, hessenARCHÄOLOGIE. The co-author Sabine Schade-Lindig are also the responsible curator.
Specimen deposition	The specimens are deposite at the Landesamt für Denkmalpflege Hessen, hessenARCHÄOLOGIE
Dating methods	The samples were dated on the basis of the archaeological evidence and using the radiocarbon method. The radiocarbon dating is also described in the Material and Methods.

☒ Tick this box to confirm that the raw and calibrated dates are available in the paper or in Supplementary Information.

Ethics oversight	No ethics vote is necessary

Note that full information on the approval of the study protocol must also be provided in the manuscript.
